# The Role of Environmental Factors in the Development of Celiac Disease: What Is New?

**DOI:** 10.3390/diseases3040282

**Published:** 2015-10-27

**Authors:** Elena Lionetti, Carlo Catassi

**Affiliations:** 1 Department of Pediatrics, University of Catania, Via S. Sofia 78, 95124 Catania, Italy; 2 Department of Pediatrics, Marche Polytechnic University, Ancona, Via Corridoni 11, 60123 Ancona, Italy; E-Mail: c.catassi@univpm.it; 3 The Division of Pediatric Gastroenterology and Nutrition and Center for Celiac Research, MassGeneral Hospital for Children, 55 Fruit Street, Boston, MA 02114, USA

**Keywords:** breastfeeding, celiac disease, gluten introduction, intestinal infections, prevention

## Abstract

Celiac disease (CD) is a systemic immune-mediated disorder caused by the ingestion of gluten-containing grains in genetically susceptible persons. It is one of the most common lifelong disorders, affecting approximately 1% of the general population. The prevalence of CD has increased in developed countries over recent decades, pointing to the role of additional environmental triggers other than gluten. It has been hypothesized that intestinal infections, the amount and quality of gluten, the intestinal microbiota, and early nutrition are all possible triggers of the switch from tolerance to an immune response to gluten. Two recent randomized controlled trials have been performed to clarify the relationship between the age at which gluten is introduced to a child’s diet and the risk of CD, showing that timing of gluten introduction does not modify the risk of CD. Both trials also showed that breastfeeding compared with no breastfeeding or breastfeeding duration or breastfeeding during gluten introduction have no effect on the risk of CD. The two trials, although not designed to address this issue, have shown that intestinal infections seem not to influence the risk of CD. Further studies are still needed to explore the missing environmental factors of CD for future prevention.

## 1. Introduction

Celiac disease is a systemic immune-mediated disorder caused by the ingestion of gluten-containing grains (wheat, rye, and barley) in genetically susceptible persons. It is one of the most common lifelong disorders on a worldwide basis, affecting approximately 1% of the general population [1,2]. The frequency of celiac disease has increased in developed countries over the last decades, a finding that points out the causal role of possible environmental triggers additional to gluten [3].

## 2. The Complex Pathogenesis of Celiac Disease: From Genes to Environment

The development of celiac disease is determined by both environmental and genetic factors. Multiple lines of evidence favoring a genetic contribution to the pathogenesis of celiac disease have been suggested by epidemiologic data: a familial aggregation is found in 5%–15% of celiac disease patients and a striking 83%–86% concordance rate was observed among monozygotic twin pairs [4]. The genetic determinants that confer susceptibility to the disease are, however, not yet fully understood. The most important genetic factor identified is the human leukocyte antigen (HLA) locus. The HLA-DQ2 (DQA1*0501-DQB1*0201) haplotype is expressed in the majority of affected patients (90%), the DQ8 haplotype (DQA1*0301-DQB1*0302) is expressed in 5%, and 5% carry at least one of the two DQ2 alleles (usually the DQB1*0201) [5]. An increased risk of celiac disease has been observed among persons who carry two DQB1*02 alleles [6,7]. The ability of these alleles in conferring individual susceptibility to celiac disease is related to their peculiar capacity to bind negatively charged peptides such as gliadin peptides resulting from the deamidation of gluten by the antitransglutaminase. The HLA antigen results in the activation of T lymphocytes, whose secretion products play a key role in causing mucosal lesions [6]. The associations found in non-HLA genome-wide linkage and association studies are much weaker. This might be because a large number of non-HLA genes contributes to the pathogenesis of celiac disease [8]. Hence, the contribution of a single predisposing non-HLA gene might be quite modest [4].

Gluten is the environmental factor required to trigger the disease, but other factors may be involved in a model of a complex multifactorial disease [7]. The total prevalence of celiac disease has, indeed, doubled in Finland during the last two decades (1.05% in 1978–1980 and 1.99% in 2000–2001) [9], and the increase cannot simply be attributed to a better rate of detection. Recently, it has been shown that celiac disease autoimmunity doubled between 1974 (one in every 501 subjects) and 1989 (one in every 219 subjects) within an American population followed since 1974. This trend apparently continued in the following years. In a different sample of the adult American population in 2001, a celiac disease prevalence of one in 105 subjects was reported. Therefore, during the last 30 years, the prevalence of celiac disease among adults in the US appeared to increase by five-fold, doubling approximately every 15 years [10]. According to the “hygiene hypothesis,” the “cleaner” environment found nowadays in developed countries led to a lower frequency of early childhood infections and differences in the spectrum of microorganisms populating the gut. These changes could modify the immune response and be responsible for a higher risk of different autoimmune disorders such as celiac disease [11]. However, the rising prevalence of adult onset of celiac disease that was observed in the US study can hardly be explained by hygienic changes occurring in childhood. The reasons for these changes are still unclear, but have to do with the environmental components of celiac disease, since genetic changes are too slow to drive these phenomena [3].

It has been recently hypothesized that all the following environmental factors are possibly involved in the switches of the tolerance–immune response balance: the amount and the quality of ingested gluten, the type and duration of wheat dough fermentation, early infant feeding, the spectrum of intestinal microorganisms and how they change over time, intestinal infections, and stressors in general [7]. However, more research is needed to determine whether and how these factors can cause loss of gluten tolerance. Recent randomized control trials have been performed to clarify the role of some of these factors on the later development of celiac disease. In the present review we provide an overview of the recent research in this field.

## 3. The Role of Infant Feeding on the Development of Celiac Disease

### 3.1. Age at Gluten Introduction

The introduction of gluten at six months of age is a long-standing practice, and although it is a rule deeply rooted in many developed countries, the optimal time of introduction of gluten in the diet of a child had never been rigorously tested. In clinical practice, many pediatricians believed that the introduction of gluten in the diet of children who have a family risk of celiac disease should be delayed to allow the maturation of the intestinal barrier and the immune response. However, investigations following the real “epidemic” of celiac disease that occurred in Sweden during the 1980s and 1990s showed that the introduction of a small amount of gluten during breastfeeding between four and six months of age reduced the risk of the disease. These data provided the basis for the hypothesis of the so-called “window of tolerance”, according to which there would be a window of time, between four and seven months of age, during which the introduction of gluten could facilitate the induction of tolerance [12,13]. The concept of the “window of tolerance” gained popularity in 2005 with the findings of a US study reporting that children at genetic risk for type 1 diabetes exposed to gluten between four and six months of age had a reduced risk of celiac disease compared to those exposed to gluten before four and after seven months of age; it is worth noting that the number of patients in this study with a diagnosis of celiac disease confirmed by intestinal biopsy was very small [14,15]. Later, a German study showed that children with a family risk of diabetes type 1 whose first dietary exposure to gluten occurred after the age of six months had an increased risk of celiac autoimmunity [16]. However, an epidemiological survey carried out in 2013 on a large Norwegian population (324 cases with celiac disease and a cohort of 81,843 healthy controls), challenged all previous observations; in particular, the results of the Norwegian study demonstrated that: (a) the introduction of gluten during breastfeeding was not protective; (b) only the delayed introduction of gluten (>6 months), but not early introduction (<4 months), was associated with an increased risk of celiac disease [17]. The main limitation of the Norwegian study was that the analysis included only children with a clinical diagnosis of celiac disease; therefore, this result could not necessarily be applied to the overall celiac population (which is at least three times larger). Another problem of this and previous case-control studies was the lack of an intervention arm.

Two randomized controlled trials have been recently performed to finally clarify the relationship between the age at which gluten is introduced to a child’s diet and the risk of celiac disease [18,19]. The Risk of Celiac Disease and Age at Gluten Introduction (CELIPREV) trial is a multicenter, prospective intervention trial comparing early (six months) and delayed (12 months) introduction of gluten in infants with a familial risk of celiac disease, followed from birth until 10 years of age [18]. Infants who had a familial risk of celiac disease (*i.e.*, infants who had at least one first-degree relative with celiac disease) were recruited in 20 centers in Italy between 2003 and 2008. Infants were randomly assigned to one of the two groups: those in group A were introduced to gluten-containing foods (pasta, semolina, and biscuits) at six months of age, and those in group B at 12 months of age. The main objective was to compare the prevalence of celiac disease according to the time of gluten introduction at five years of age. The percentage of children with celiac disease at five years of age was the same in group A and in group B (16% and 16%, *p =* 0.78); at 10 years there was still no significant difference between the two groups (hazard ratio at 10 years: 0.9; 95% Confidence Interval (CI): 0.6–1.4; *p* = 0.79). However, the average age at which celiac disease developed was 26 months in group A and 34 months in group B (*p* = 0.01). It is worth noting that in children with the high-risk HLA genotype (characterized by homozygosis for HLA-DQ2), the prevalence of celiac disease was higher in group A than in group B at all ages, although the difference was not significant (*p* = 0.51), probably due to the small size of the sample of children with this genotype. During follow-up, complications related to celiac disease (*i.e.*, autoimmune thyroid diseases, type 1 diabetes, or both) did not develop in any child from the two groups. Therefore, the CELIPREV study has shown that postponing the introduction of gluten to 12 months of age has no effect on the risk of developing the disease in the long term: it does not reduce or increase the risk of disease. However, delaying the introduction of gluten had two potentially positive consequences: (a) to delay the development of the disease; and (b) to possibly reduce the frequency of celiac disease in the HLA-high-risk group. In fact, although this last effect did not reach statistical significance, it is worthy of further investigations. The hypothesis of the “window of tolerance” was therefore not supported, as there was no difference in the risk of celiac disease among children who were introduced to gluten at six months (during the “window” open) and those who were introduced to gluten at 12 months (when the “window” was closed).

The European multicenter project Prevent Celiac Disease (PREVENT-CD) trial is a multicenter, randomized, double-blind, placebo-controlled dietary intervention study aimed at comparing the introduction of small quantities of gluten (100 mg/daily) from 16 to 24 weeks of age with a placebo, followed by standard gluten consumption after the intervention in infants with a familial risk of celiac disease, followed from birth to at least three years of age [19]. The results of this trial demonstrated that the introduction of small amounts of gluten during the window of tolerance does not reduce the risk of the disease. The cumulative incidence of celiac disease among patients three years of age was indeed similar in the gluten group and the placebo group (5.9% and 4.5%, respectively; hazard ratio at three years: 1.23; 95% CI, 0.79 to 1.91, *p* = 0.47).

### 3.2. Breastfeeding

A protective role of breastfeeding against celiac disease has long been perceived, mostly based on some retrospective studies [20,21,22,23] and a systematic review of the literature [24] and a meta-analysis that summarized those results [25]. The Norwegian epidemiological investigation had instead shown that breastfeeding did not exert any protective effect against the development of celiac disease; the average duration of breastfeeding was, indeed, even longer in children with celiac disease (10.4 months) compared with controls (9.9 months), and the risk of disease was significantly higher in babies breastfed for more than 12 months [17].

**Figure 1:**
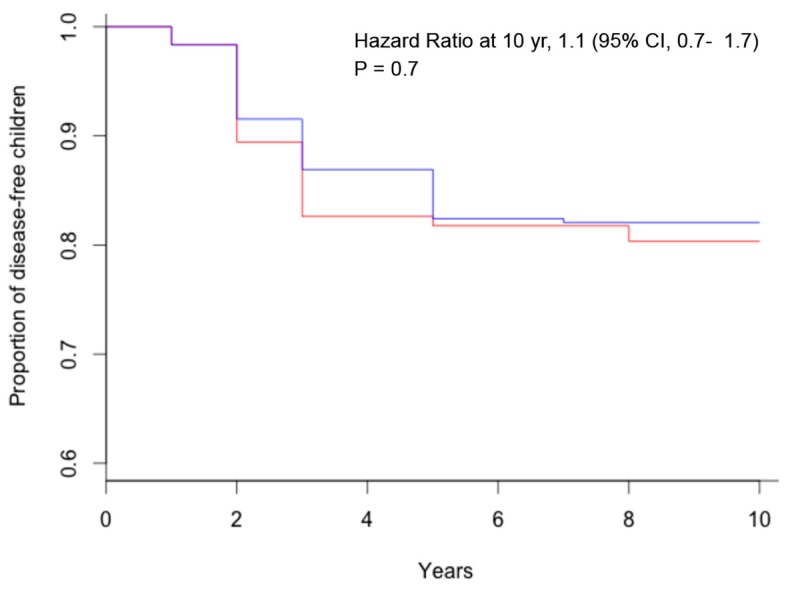
Kaplan–Meier estimates of celiac disease, according to breastfeeding (red = no breastfeeding; blue = any breastfeeding).

**Figure 2:**
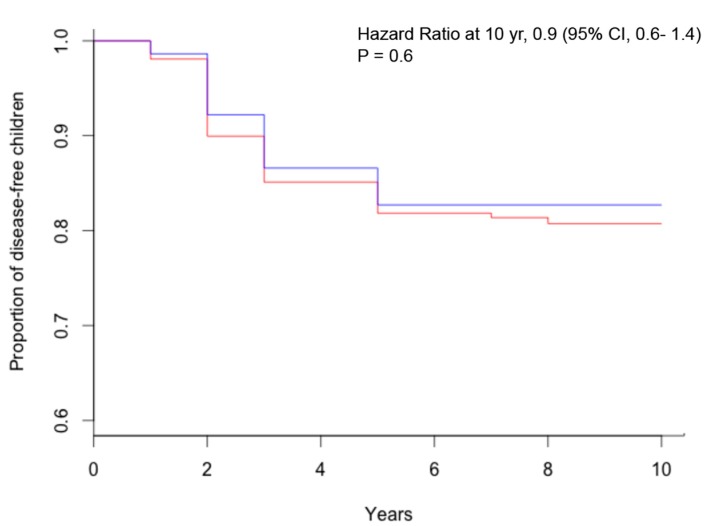
Kaplan–Meier estimates of celiac disease, according to breastfeeding duration (red = breastfeeding < 6 months; blue = breastfeeding ≥ 6 months).

**Figure 3:**
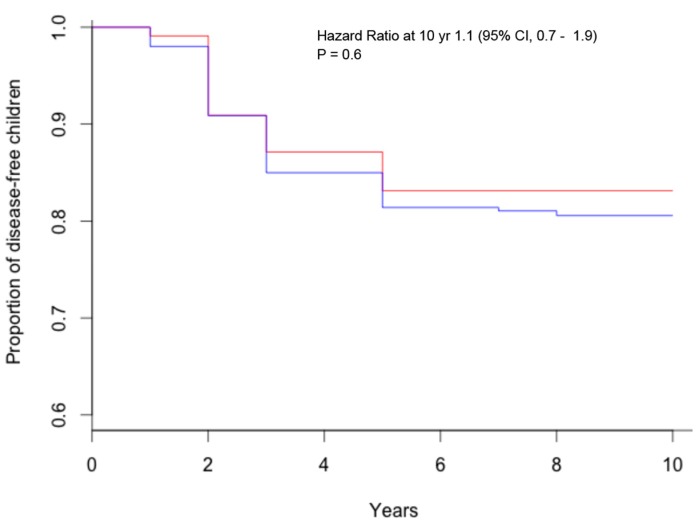
Kaplan–Meier estimates of celiac disease, according to breastfeeding during gluten introduction (red = breastfeeding during gluten introduction; blue = no breastfeeding during gluten introduction).

The study CELIPREV brought a clarification on this important aspect of child nutrition. In the cohort of infants at familial risk of celiac disease, no protective effect of breastfeeding for the development of celiac disease was observed: the average duration of breastfeeding was similar for children that developed celiac disease and for those that did not develop celiac disease (5.6 and 5.8 months, respectively); there was no significant difference in the percentage of children that developed celiac disease among children breastfed and those never breastfed (Figure 1) and among those breastfed for more or less than six months (Figure 2); finally, there was no significant difference in the percentage of children that developed celiac disease among children that were introduced gluten during breastfeeding and in those that were introduced gluten without breastfeeding (Figure 3) [18]. The same results were reported by the PREVENT-CD study [19]. Therefore, although there are many good reasons to recommend prolonged breastfeeding of newborns, prospective studies have not observed a protective effect against celiac disease.

### 3.3. Amount and Type of Gluten

The PREVENT-CD study showed that the introduction of gluten at a dose of 200 mg/day at four to six months of age compared to the avoidance of gluten does not have an effect on the risk of celiac disease at three years of age [19]. There are no data on other amounts of gluten introduced at weaning, nor on the type of gluten introduced and later celiac disease development from the prospective interventional trial. Therefore, based on the current literature evidence, no conclusion can be made from these results.

## 4. Intestinal Infections and Risk of Celiac Disease

Intestinal infections (such as rotavirus among infants and Campylobacter among adults) have recently been shown to be associated with an increased risk of celiac disease [26,27]. Infections might change gut permeability, leading to the passage of immunogenic gluten peptides through the epithelial barrier. The other possibility implies that sequence similarities exist between proteins produced during rotavirus infections and proteins of gluten. Infections, especially viral infections, may also alter mucosal gene expression, specifically the expression of pattern recognition receptors, which are considered to be in a key position to determine host-environment interactions [28,29,30]. The area has not been studied much with enterocytes, but studies with other cell lines give indicative insights. Altered mucosal gene expression of tool-like receptors and their regulators has been, indeed, reported in pediatric celiac disease subjects [31].

On the other hand, rates of intestinal infections have not increased markedly in recent decades, and thus are not likely to be driving the epidemic of celiac disease. In contrast, a lack of exposure to certain microbes is the hallmark of modern times [32]. The CELIPREV trial, although not designed to address this outcome, evaluated the role of all nutritional and genetic variables studied (breastfeeding, amount of gluten, age at gluten introduction, genotype, gender, celiac disease-affected first-degree relative, and intestinal infections) in influencing the risk of celiac disease by a data-mining approach with decision-tree induction. Interestingly, none of the variables studied, including intestinal infections, had a significant effect in predicting the development of celiac disease, with the exception of the HLA genotype. Indeed, the risk of celiac disease was far higher among children with high-risk HLA than among those with standard-risk HLA (26% *vs.* 16%, *p* = 0.05), confirming that predisposing HLA gene dosing is, to our knowledge, the most influential variable in increasing the risk of developing celiac disease [18]. Also, the PREVENT-CD trial evaluated the cumulative incidence of celiac disease according to the presence of gastrointestinal infection in the first 18 months of life, showing no effect on the risk of celiac disease (4.9% *vs.* 6.4%, *p* = 0.61). Moreover, the authors also found no difference in the cumulative incidence of celiac disease when comparing children that were administered a rotavirus vaccination *versus* those that were not administered the vaccination (2.5% *vs.* 6.0%, *p* = 0.96) [19].

## 5. Intestinal Microbiota and Host-Microbiota Interactions in Celiac Disease

There is now accumulating evidence that gut microbiota plays an important role in the regulation of intestinal immune responses and in the maintenance of intestinal homeostasis. Most observational studies in children and adults have shown intestinal dysbiosis (*i.e.*, altered gut microbiota composition or function) in celiac disease patients, untreated and treated with a gluten-free diet, compared to healthy controls [33,34,35,36,37,38,39]. Celiac disease patients with gastrointestinal symptoms are also known to have a different microbiota compared to patients with dermatitis herpetiformis and controls, suggesting that the microbiota is involved in disease manifestation [40]. Furthermore, a dysbiotic microbiota seems to be associated with persistent gastrointestinal symptoms in treated celiac disease patients, suggesting its pathogenic implication in these particular cases [41]. Nevertheless, other authors report no differences in mucosa-associated duodenal microbiome composition and diversity in celiac disease children as compared to controls [42], and there is lack of consensus and understanding of what constitutes a CD-promoting microbiota [33].

Dysbiosis may be the result of both genetic and environmental factors. Specific host-genetic makeup could promote the colonization of pathobionts and reduce symbionts, thus leading to dysbiosis [33]. The genotype of infants at family risk of developing celiac disease, carrying the HLA-DQ2 haplotypes, has indeed been shown to influence the early gut microbiota composition, suggesting that a specific disease-biased host genotype may select for the first gut colonizers and could contribute to determining disease risk [43]. Recent studies also showed a significant association of celiac disease with homozigosity for a nonsense mutation in the fucosyltransferase 2 non-secretor status, which has been shown to be a major determinant for the gut microbial spectrum [44]. The non-secretor individuals were demonstrated to have an altered mucosa-associated microbiota in their intestinal tract, characterized by reduced diversity, richness, and abundance of *Bifidobacterium spp.*, a bacterial genus that may play an important role in autoimmune disease risk [45,46]. Besides host genetics, environmental factors could also influence microbiota composition; indeed, the milk-feeding type (breast-milk *vs.* formula), the mode of delivery, antibiotics, and other drugs are also well-known environmental factors exerting a profound impact on the microbiota composition, potentially modifying its functional role in health and disease [33,47].

Intestinal dysbiosis might contribute to the etiopathogenesis of celiac disease either by itself providing proteolytic activities that influence the generation of toxic and immunogenic peptides from gluten and by mediating host-microbe interactions, which could influence the intestinal barrier and immune function (e.g., via regulation of the cytokine network of pro-inflammatory and anti-inflammatory factors) [33,34]. Nonetheless, it still remains unclear whether the changes in the microbiota are a cause or a secondary consequence of celiac disease development. Animal models, including germ-free and gnotobiotic models, will be of critical value to study whether the composition of the gut microbiota influences the loss of tolerance to gluten in genetically susceptible hosts and the mechanisms through which microbes can influence host responses to gluten. These types of studies will be helpful in determining causality, but their direct translational value might be limited due to the associated limitations with animal models of celiac disease. Thus, clinical studies will be needed to provide translational value of basic studies [34]. Unfortunately, neither CELIPREV nor PREVENT-CD has investigated this intriguing issue in a clinical setting. Prospective studies in healthy infants at family risk of celiac disease are underway to decipher the co-evolution of the gut microbiome and the host genome in response to environmental factors and the possible causal relationships with celiac disease onset.

## 6. Conclusions

As stated in the editorial published on this issue, “we are still in search of environmental factors that may affect the development of the disease” and “the two trials—CELIPREV and PREVENT-CD—are today a starting point rather than an end point of the research in this field” [48].

A revision of the current recommendations of the European Society of Gastroenterology, Hepatology and Pediatric Nutrition (ESPGHAN) on weaning [49], which recommend the introduction of gluten from four to seven months of age, during the “window of tolerance”, and the introduction of gluten while the infant is still being breastfed in order to reduce the risk of celiac disease is strongly advised. Evidence from recent randomized controlled trials showed that neither breastfeeding nor breastfeeding during gluten introduction may reduce the risk of celiac disease, and that the age of gluten introduction into the infant's diet, whether early or late, does not influence the risk of celiac disease during childhood, and, thus, indicates that primary prevention of CD is not possible at the present time

Further studies are needed to determine whether other environmental factors, such as the composition of the intestinal microbiota, the mode of delivery, the metabolic profile, the vaccination program, and the use of antibiotics or other drugs actually influence the balance between the immune response and tolerance to gluten. Also, the role of intrauterine and perinatal exposure is still to be explored. Moreover, the reduction of gluten antigenicity and/or the elimination of toxic peptides from gluten could be a workable strategy of primary prevention: the modifications of gluten antigenicity may be achieved in several manners, from the selection of natural cereal cultivars to the genetic modification of gluten peptide sequences or, more likely, producing genetically modified organisms, in which toxic sequences are deleted or silenced [50]. The results of all these studies could be instrumental in determining how to prevent not only the onset of celiac disease but also other autoimmune disorders.
